# Border Security: The Role of RIPK3 in Epithelium Homeostasis

**DOI:** 10.3389/fcell.2016.00070

**Published:** 2016-06-28

**Authors:** Kenta Moriwaki, Sakthi Balaji, Francis Ka-Ming Chan

**Affiliations:** Department of Pathology, Immunology and Microbiology Program, University of Massachusetts Medical SchoolWorcester, MA, USA

**Keywords:** DSS, RIPK1, necroptosis, inflammasome, IL-1β, IL-23, tissue repair, cancer

## Abstract

Receptor interacting protein kinase 3 (RIPK3) is a crucial inducer of necroptosis. Its activity is controlled by interaction with other signal adaptors through the “RIP homotypic interaction motif” (RHIM). Recent studies revealed a critical function for RIPK3 in the maintenance of epithelial tissue integrity. In mice with genetic deficiency of the apoptosis adaptors FADD or caspase 8, RIPK3 promotes necroptotic cell death of epithelial cells, leading to excessive and lethal inflammation. In contrast, when FADD and caspase 8 functions are intact, RIPK3 serves as a protector of intestinal epithelial integrity by promoting injury-induced wound repair. In the latter case, RIPK3 promotes optimal cytokine expression by cells of hematopoietic origin. Specifically, bone marrow derived dendritic cells (BMDCs) have an obligate requirement for RIPK3 for optimal secretion of mature IL-1β and other inflammatory cytokines in response to toll-like receptor 4 (TLR4) stimulation. RIPK3 promotes cytokine expression through two complementary mechanisms: NF-κB dependent gene transcription and processing of pro-IL-1β. We propose that RIPK3 functions in different cell compartments to mediate inflammation through distinct mechanisms.

## Introduction

Epithelial tissues are the first lines of defense against pathogens and environmental insults. Genetic experiments in mice show that many adaptors of the TNF receptor pathway have critical functions in maintenance of epithelial integrity in the skin and intestine. The receptor interacting protein kinases (RIPKs) are critical regulators of cell death and inflammatory signaling by TNF-like death receptors, toll-like receptor 3 (TLR3) and TLR4 (Chan et al., 2015; Pasparakis and Vandenabeele, 2015). RIPK1 and RIPK3, the two key kinases that regulate cell death by these receptors, have important functions in regulating inflammation at epithelial surfaces. Despite their well-known roles in cell death, emerging evidence indicates that RIPK1 and RIPK3 are key protectors of epithelial integrity. Another surprising realization is that rather than acting in synergy, these two kinases can sometimes exert opposing effects in epithelial tissues. Here, we will discuss recent studies that led to these emerging themes. We will also provide some experimental data from an intestinal injury model to illustrate the influence of commensal microbiota on RIPK3-mediated tissue protection and the long-term consequence of RIPK3 deficiency on chronic injury-induced inflammation.

## RIPK1 and RIPK3 mediate TNF-induced cell death signals

**Figure 1:**
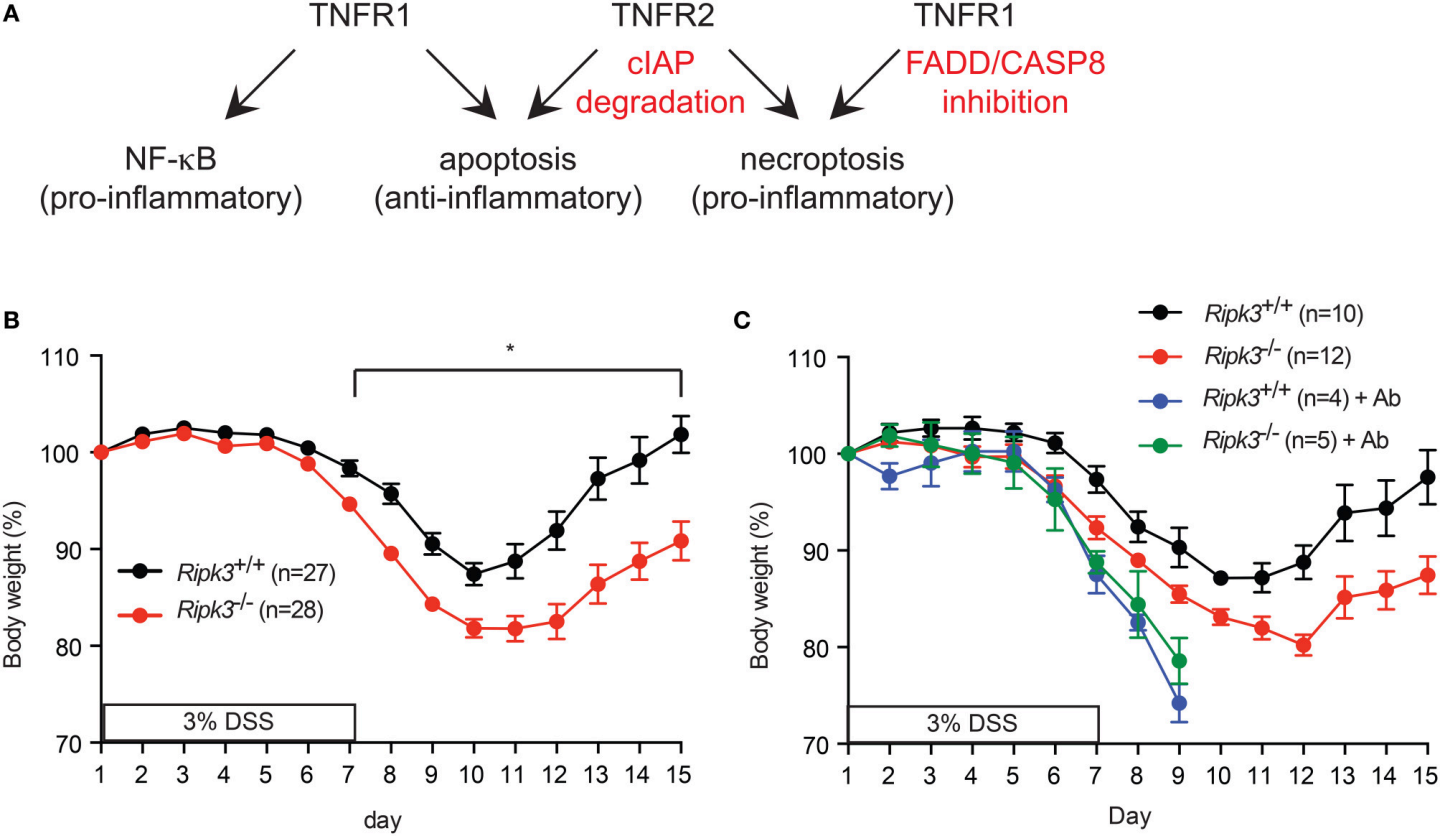
**(A)** Signaling by TNFR1, TNFR2, and the availability of FADD, Caspase 8 and cellular IAPs determine the signaling outcome of TNF. **(B–C)** RIPK3 controls DSS-induced colitis is dose- and commensal microbiota-dependent. **(B)** Change in body weight of mice treated with **(A)** 3% DSS-containing drinking water. **(C)** Mice were treated with antibiotics cocktail (Ab) for 4 weeks prior to treatment with 3% DSS. Body weight loss was normalized to that on day 1. ^*^p < 0.001.

TNF is a pleiotropic cytokine that exerts its biological effects through binding to two distinct cell surface receptors, TNFR1 (p55/p60) and TNFR2 (p75/p80). TNFR1 is constitutively expressed in most cells, while TNFR2 expression is inducible in response to activation signals (Kim and Teh, 2004). While most of the biological effects of TNF can be attributed to the action of TNFR1, genetic evidence suggests that TNFR2 can sometimes function independently of TNFR1 (Kim and Teh, 2001, 2004; Dayer Schneider et al., 2009; Kim et al., 2009). Despite the name tumor necrosis factor, TNF predominantly stimulates gene transcription through the MAP kinase and NF-κB pathways. The NF-κB pathway downstream of TNFR1 has been extensively studied and has a pivotal role in expression of pro-survival factors such as the cellular FLICE (aka caspase 8)-like inhibitor protein (cFLIP) and the cellular inhibitor of apoptosis protein 1 (cIAP1) and cIAP2. Since TNFR2 stimulates proteasomal degradation of the E3 ligases cIAP1 and cIAP2 and the adaptor protein TRAF2 [(Csomos et al., 2009) and unpublished results], cells that express TNFR1 and TNFR2 are much more sensitive to TNF-induced apoptosis than cells expressing only TNFR1 (Chan and Lenardo, 2000). Apoptosis induced in the absence of the cIAPs requires RIPK1 kinase activity (Wang et al., 2008). When caspase 8 activity is inhibited, TNF stimulates “necroptosis,” a form of regulated necrosis marked by rapid loss of plasma membrane integrity. TNF-induced necroptosis requires activation of the RIPK1-RIPK3-mixed lineage kinase domain-like (MLKL) axis (Chan et al., 2015). Because of the leakage of cellular damage-associated molecular patterns (DAMPs) from necroptotic cells, necroptosis is a more inflammatory form of cell death than apoptosis (Aaes et al., 2016). Thus, TNF can stimulate inflammation through at least two distinct mechanisms, NF-κB-dependent gene expression and necroptosis (Figure 1A).

## Genetic ablation of FADD or caspase 8 primes epithelial cells to necroptosis

Both RIPK1 and RIPK3 are cleavage substrates of caspase 8 (Lin et al., 1999; Feng et al., 2007). Cleavage of RIPK1 and RIPK3 inactivates their pro-necrotic activity (Cho et al., 2009; He et al., 2009). Hence, necroptosis is optimally induced when caspase 8 or its adaptor FADD is inhibited (Figure 1A). This yin-yang relationship between caspase-dependent apoptosis and RIPK-mediated necroptosis is highlighted during embryonic development in which genetic deletion of FADD or caspase 8 led to embryonic lethality on E10.5 (Yeh et al., 1998; Zhang et al., 1998). This developmental defect is corrected by crossing the mice to *Ripk1*^−∕−^ or *Ripk3*^−∕−^ mice (Kaiser et al., 2011; Oberst et al., 2011; Zhang et al., 2011), although *Fadd*^−∕−^*Ripk1*^−∕−^ mice still suffered from postnatal lethality due to other RIPK1-associated survival functions (Zhang et al., 2011; Dillon et al., 2014; Kaiser et al., 2014; Rickard et al., 2014). Tissue specific deletion of FADD or caspase 8 also led to spontaneous and fatal inflammation in the respective tissues (Kovalenko et al., 2009; Bonnet et al., 2011; Gunther et al., 2011; Welz et al., 2011). The inflammation was marked by necrotic cell death and an increase in inflammatory cytokine expression. Again, the disease was reversed by deletion of *Ripk3*. These results led to the popular view that RIPK3-mediated necroptosis is a key driver of inflammation in epithelial tissues.

## RIPK3 facilitates wound repair in epithelial tissues

Because loss of FADD or caspase 8 compromises organismal survival, it is unlikely to be a natural occurrence. We therefore, questioned whether RIPK3 has similar pro-inflammatory function in epithelial homeostasis when FADD and caspase 8 functions are intact. We tested this notion using dextran sodium sulfate (DSS) to induce intestinal injury in *Ripk3*^−∕−^ mice. DSS causes intestinal injury through unknown mechanisms, leading to inflammation that bears some resemblance to human colitis (Wirtz et al., 2007). The inflammatory response is essential to initiate repair of the damaged intestinal epithelium. Surprisingly, we found that *Ripk3*^−∕−^ mice were more sensitive to DSS compared with their littermates (Moriwaki et al., 2014). The hypersensitivity of *Ripk3*^−∕−^ mice was dose-dependent, since it was observed with 3% DSS, but not at lower doses of DSS (Figure 1B and see below). Moreover, the duration of DSS treatment and potency of different batches of DSS can influence the severity of disease (unpublished observation). In addition, DSS-induced colitis is highly dependent on commensal microbiota. For example, antibiotics treatment can exacerbate DSS-induced colitis by expansion of certain resistant E. coli pathobiont that stimulates the Naip5-Nlrc4 inflammasome (Ayres et al., 2012). Consistent with these observations, antibiotics treatment led to equally severe DSS-induced colitis in *Ripk3*^−∕−^ and wild type littermates (Figure 1C). Because of the severe diarrhea and body weight loss in the antibiotics-treated mice, all the animals had to be euthanized on day 9. These results suggest that RIPK3 may facilitate sensing of specific bacterial pathogen-associated molecular patterns (PAMPs). These factors can all affect the outcome of DSS-induced colitis and explain why a recent study did not find Ripk3^−∕−^ mice to be more sensitive to DSS (Newton et al., 2016).

**Figure 2:**
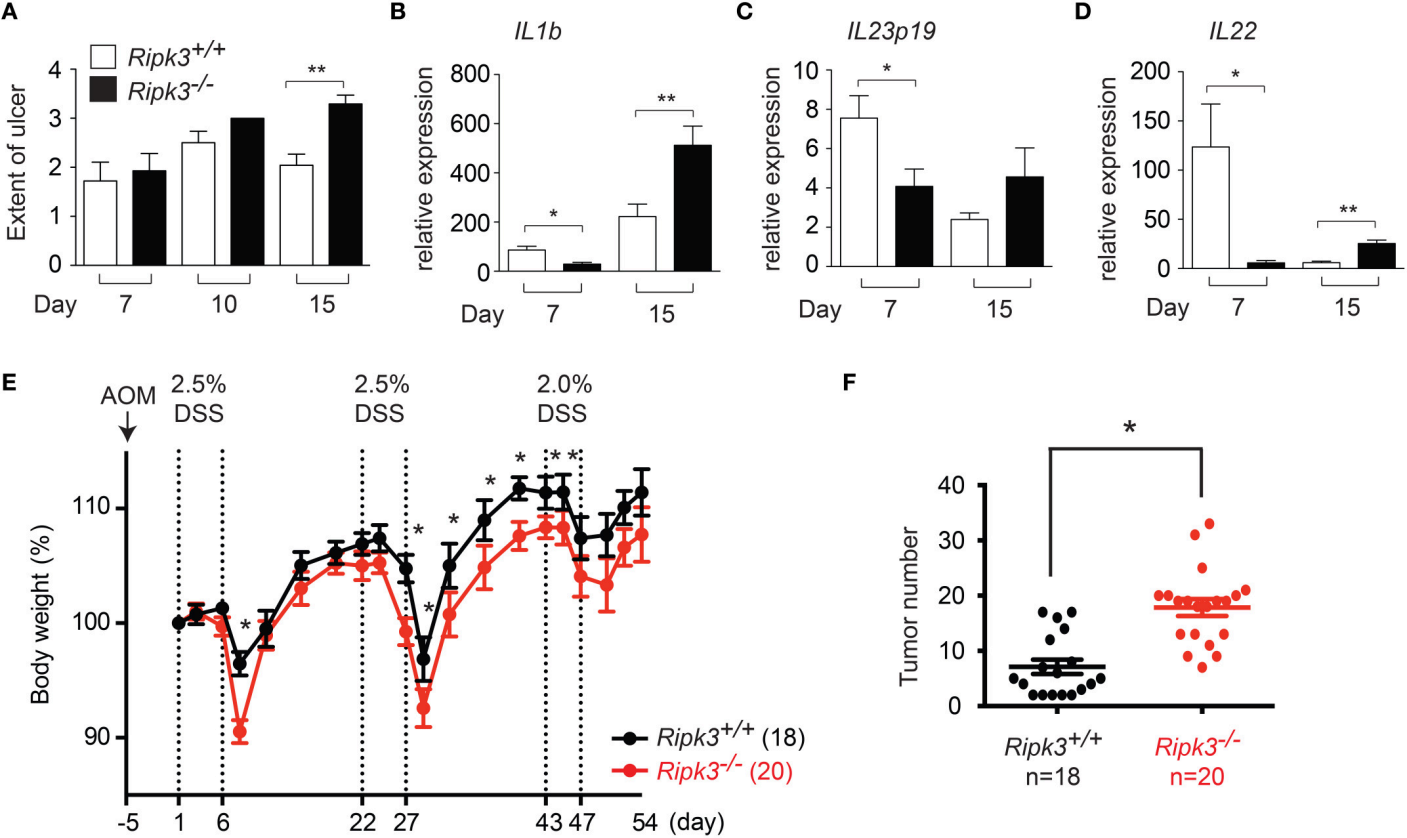
**Sustained injury in ***Ripk3***^**−∕−**^ mice led to chronic elevated cytokine expression**. **(A)** Cell injury in the intestine of DSS-treated mice was quantified by blind histology scoring. *n* = 7–12. **(B–D)** Expression of **(B)** *Il1b*, **(C)** *Il23p19*, **(D)** *Il22* in DSS-treated *Ripk3*^−∕−^ colon exhibit biphasic pattern of expression. *n* = 4–11. ^*^p ≤ 0.05, ^**^p < 0.01. **(E–F)** *Ripk3*^−∕−^ mice are more susceptible to inflammation-induced colorectal cancer. **(E)** *Ripk3*^+∕+^ and *Ripk3*^−∕−^ mice were treated with AOM and three cycles of DSS as indicated. Change in body weight of the mice is shown. Body weight loss was normalized to that on day 1. **(F)** The number of tumors in the whole colon was counted on day 54. ^*^p < 0.0001.

We previously reported that epithelial cell injury was similar in wild type and *Ripk3*^−∕−^ mice 4 days after DSS treatment (Moriwaki et al., 2014). However, intestinal epithelial cell (IEC) proliferation was severely impaired in *Ripk3*^−∕−^ mice (Moriwaki et al., 2014), indicating that RIPK3 protects against DSS-induced injury through promoting IEC regeneration and tissue repair. To determine the long-term impact of impaired IEC regeneration, we tracked tissue injury through day 15 post-DSS treatment. To allow evaluation of tissue repair, the mice were returned to normal drinking water on day 8. Under these conditions, IEC injury continued to rise between day 7 and 10, but was reduced by day 15 in wild type littermates (Figure 2A). By contrast, IEC injury was sustained at elevated level in *Ripk3*^−∕−^ mice on day 15 (Figure 2A). These results indicate that impaired wound repair led to sustained tissue injury in *Ripk3*^−∕−^ mice.

Sustained injury could lead to chronic elevated cytokine expression. We therefore, examined the long-term impact of failed wound repair on cytokine expression. Consistent with our previous report (Moriwaki et al., 2014), expression of the repair-associated cytokines IL-1β, IL-23, and IL-22 was reduced in *Ripk3*^−∕−^ mice on day 7 (Figures 2B–D). By contrast, expression of these cytokines was elevated in *Ripk3*^−∕−^ mice on day 15 (Figures 2B–D). Although we cannot rule out contribution from other inflammatory mediators, these results are consistent with the notion that chronic injury caused by RIPK3 deficiency eventually led to sustained production of inflammatory cytokines in *Ripk3*^−∕−^ mice. In this regard, it is interesting to note that elevated RIPK3 expression was detected in human patients of inflammatory bowel diseases (Pierdomenico et al., 2014). This suggests that RIPK3 may similarly drive inflammatory cytokine expression in human inflammatory bowel diseases.

Chronic inflammation can promote tumorigenesis. We therefore, asked if DSS-treated *Ripk3*^−∕−^ mice are more susceptible to developing colon carcinoma. To this end, we treated mice with a single dose of the carcinogen azoxymethane (AOM), followed by three cycles of 2.5% DSS treatment (Figure 2E). With the lower dose of DSS, the body weight change was much more comparable between *Ripk3*^−∕−^ mice and their wild type littermates (Figure 2E), indicating that RIPK3-mediated tissue repair response is dose-dependent. We found that the number of colon tumors was significantly increased in *Ripk3*^−∕−^ mice (Figure 2F). These results therefore indicate that the loss of RIPK3 promotes inflammation-induced tumorigenesis in the colon. In contrast, a recent report shows that the RIPK1-RIPK3 necrosome promotes pancreatic oncogenesis through upregulation of the chemokine CXCL1 (Seifert et al., 2016). It is noteworthy that many breast and colon tumors do not express RIPK3 (He et al., 2009; Moriwaki et al., 2015b), while pancreatic cancers often exhibit increased RIPK1 and RIPK3 expression (Seifert et al., 2016). These results therefore highlight the tissue-specific effects of RIPK3 in controlling inflammation and tumorigenesis.

The role of RIPK3 in wound repair is not restricted to intestinal tissues. RIPK3 expression was induced during cutaneous wound healing (Adams et al., 2007), and *Ripk3*^−∕−^ mice exhibited delayed healing of dorsal cutaneous wound (Godwin et al., 2015). In this model, *Ripk3*^−∕−^ mice first exhibited reduced cytokine expression, followed by elevated cytokine expression later in the reaction. Hence, a similar biphasic cytokine expression pattern was observed in *Ripk3*^−∕−^ mice in response to intestinal and cutaneous injury. Consistent with a role for RIPK3 in wound repair, caspase 8, the natural regulator of RIPK3 activity, has also been implicated in cutaneous wound healing (Lee et al., 2009; Li et al., 2010). Based on these results, RIPK3 is likely to have a broader role in maintenance of epithelial integrity.

## RIPK3 promotes intestinal barrier repair though necroptosis-independent mechanisms

Recent evidence indicates that RIPK3 also has important functions in NF-κB and NLRP3 inflammasome activation. Indeed, these non-necroptotic activities are crucial for RIPK3-mediated tissue repair in the damaged intestine. In bone marrow derived dendritic cells (BMDCs), RIPK3 promotes nuclear translocation of the atypical NF-κB dimer RelB-p50 upon stimulation of the LPS receptor TLR4 (Moriwaki et al., 2014). This activity is distinct from that of necroptosis, since RIPK3 kinase inhibitors had no effects on LPS-induced RelB-p50 nuclear translocation (Moriwaki et al., 2014). As such, *Ripk3*^−∕−^ BMDCs were highly defective in expression of many inflammatory cytokines including IL-1β and IL-23 (Moriwaki et al., 2014, 2015a), the two key repair-associated cytokines in the DSS-induced colitis model. Importantly, RIPK3-dependent cytokine expression was only observed with GM-CSF and IL-4 differentiated BMDCs, but not with BMDCs differentiated with GM-CSF alone, bone marrow derived macrophages (BMDMs) or splenic CD11c^+^ DCs [(Moriwaki and Chan, 2014) and data not shown]. These results highlight the important concept that the outcome of RIPK3 signaling is highly context- and cell type-dependent. Identifying the relevant immune cell type in the lamina propria that mediates these protective signals during tissue injury will be of prime importance.

Besides NF-κB dependent cytokine gene expression, RIPK3 is also an important factor for pro-IL-1β processing, a function that is critical for optimal IL-1β expression in response to DSS-induced intestinal injury. The mechanism by which RIPK3 stimulates pro-IL-1β processing differs depending on the activity of caspase 8 and the IAPs. In BMDCs with intact caspase 8 activity, RIPK3 forms an atypical caspase 8 activating complex that also contains FADD and RIPK1 (Moriwaki et al., 2015a). This complex can directly cleave pro-IL-1β, or indirectly through activation of the NLRP3 inflammasome (Lawlor et al., 2015). Importantly, RIPK3 kinase activity is dispensable for activity of this complex (Moriwaki et al., 2015a). When caspase 8 and IAP activities are both inhibited, RIPK3 directly engages MLKL to stimulate NLRP3 inflammasome activation independent of RIPK1 (Kang et al., 2015; Lawlor et al., 2015). Understanding the precise mechanism by which RIPK3 activates the NLRP3 inflammasome is clearly going to be a topic of intense investigation in the near future.

## RIPK1 and RIPK3 do not function in synergy in epithelial tissues

As we have discussed already, *Ripk1* or *Ripk3* deficiency could both rescue the embryonic lethality of *Fadd*^−∕−^ or *Casp8*^−∕−^ mice. Although these genetic experiments indicate that RIPK1 and RIPK3 act in synergy to promote necroptosis, this is not the case in epithelial tissues. In fact, RIPK1 antagonizes RIPK3 in epithelial tissues to preserve barrier integrity. For example, skin epithelium-specific deletion of *Ripk1* led to spontaneous and lethal inflammation that was fully corrected by co-deletion of *Ripk3* (Dannappel et al., 2014). Similar spontaneous and lethal inflammation was observed in mice with intestinal epithelium-specific deletion of *Ripk1*. However, in contrast to the skin, *Ripk3* deletion alone was not sufficient to rescue the disease. Instead, compound deficiency of *Fadd* and *Ripk3* was required to fully inhibit the inflammatory disease (Dannappel et al., 2014; Takahashi et al., 2014). Interestingly, mice expressing a kinase inactive version of RIPK1 do not develop spontaneous colitis or skin inflammation (Berger et al., 2014; Dannappel et al., 2014; Newton et al., 2014; Takahashi et al., 2014). Since RIPK1 kinase activity is essential for its apoptotic and necroptotic effects, these results indicate that the death-inducing function of RIPK1 is not responsible for survival of epithelial tissues. Rather, RIPK1 provides a “survival scaffold” that counteracts the deleterious effects of RIPK3 through other yet-to-be defined mechanisms.

## Conclusion

RIPK3 is widely considered to be an inducer of inflammation because of its role in necroptosis. With the recent discoveries that RIPK3 can also promote NF-κB and inflammasome activation, a more balanced view of RIPK3 biology will require integration of these non-necroptotic signaling functions. Because non-necroptotic signaling by RIPK3 is cell type- and context-specific, novel mouse models such as those that will allow tissue-specific inactivation of *Ripk3* will be useful in deciphering the roles and mechanisms of RIPK3 in epithelial inflammatory diseases. Moreover, the studies in epithelial tissues implicate that a RIPK1 and RIPK3 “interactome” that is distinct from that of classical necroptosis is likely involved in the maintenance of barrier integrity. Unraveling the distinct signaling pathways in different cell compartments that mediate these effects will therefore be a critical endeavor in future investigations.

## Materials and methods

### Mice

Nine to 11 week old female *Ripk3*^−∕−^ mice and their wild type littermates on C57BL/6J background were treated with 3% DSS (MP Biomedicals, molecular mass 36,000–50,000 Da) for 7 days, followed by 8 days of regular water unless otherwise stated. For antibiotics treatment, mice were fed with drinking water containing 1 g/L neomycin, 1 g/L ampicillin, 1 g/L metronidazole, 0.5 g/L vancomycin and 5 g/L sucrose for 4 weeks prior to DSS treatment. For tumor induction, female mice (9–11 weeks) were injected intraperitoneally with AOM (Sigma) at a dose of 10 mg/kg body weight. After 5 days, mice were administered 2.5% DSS in the drinking water for 5 days followed by 16 days of regular water. This cycle was repeated twice. In the third cycle, mice were administered 2% DSS for 4 days followed by 8 days of regular water. Mice were sacrificed on day 54 and tumor number was macroscopically counted. All animal experiments were approved by the institutional animal care and use committee.

### Real-time PCR

Total RNA was extracted from colon tissues using RNeasy kit (Qiagen). cDNA was synthesized using Superscript III (Invitrogen). Real-time PCR analysis using iQ SYBR Green supermix (Bio-Rad laboratories) was performed on C1000 thermal cycler and CFX96 real-time system (Bio-Rad laboratories). Expression of cytokines was normalized to that of TATA box binding protein (TBP). Primers used: *Il1b*: 5′- CCCAACTGG TACATCAGCAC-3′, 5′-TCTGCTCATTCA CGAAAAGG-3′; *Il22*: 5′-TTGAGGTGTCCA ACTTCCAGCA-3′, 5′-AGCCGGACA TCTGTGTTGTTA-3′; *Il23p19*: 5′- CCAGCG GGACATATGAATCT-3′, 5′- AGGCTCCCCTTT GAAGATGT-3′; *Tbp*: 5′- CAAACCCAGAAT TGTTCTCCTT-3′, 5′- ATGTGGTCTTCC TGAATCCCT-3′.

## Author contributions

KM and FC conceived and designed the experiments. KM and SB performed the experiments. FC wrote the paper with assistance from KM.

## Funding

This work is supported by grants from the NIH (AI 119030) and the Crohn's and Colitis Foundation of America (326364).

### Conflict of interest statement

The authors declare that the research was conducted in the absence of any commercial or financial relationships that could be construed as a potential conflict of interest.
